# Why do we need a(nother) new data format? A standardized data format for azimuthally integrated area detector data

**DOI:** 10.1107/S1600577526004224

**Published:** 2026-04-28

**Authors:** Mads Ry Vogel Jørgensen, Frederik Holm Gjørup, Meghdad Yazdi-Rizi, Zdenek Matej, Wout De Nolf, Paul Bell

**Affiliations:** a Department of Chemistry & iNANO Aarhus University Langelandsgade 140 Aarhus C Denmark; b MAX IV Laboratory Lund University Fotongatan 2 Lund Sweden; c ESRF – The European Synchrotron 71 Avenue des Martyrs 38043Grenoble France; ESRF – The European Synchrotron, France

**Keywords:** data format, PXRD, scattering, azimuthal integration

## Abstract

Many powder X-ray diffraction, total scattering, and small-angle X-ray scattering experiments at synchrotron facilities yield azimuthally integrated intensity data, yet current practice relies on facility-specific data formats that hinder inter-operability and reuse. We propose a standardized HDF5-based format within the NeXus convention (NXazint), designed to store 1D and 2D integrated data along with essential metadata for direct use in analysis software. This approach supports FAIR principles, simplifies handling of large datasets, and promotes compatibility across beamlines and facilities. The format is already implemented at MAX IV, with visualization and powder X-ray diffraction refinement tools available.

## Abstract 2

A standardized HDF5-based data format for azimuthally integrated scattering and diffraction data, developed within the NeXus framework, is presented. This format enables inter-operability across facilities and software, helps support FAIR principles, and simplifies data analysis workflows.

If you carry out a scattering experiment at a synchrotron beamline in the future, you may notice that the data files you bring home have changed. Data formats are seldom very exciting, so why should you appreciate this new one?

The use of synchrotron radiation for powder diffraction and scattering is ubiquitous, and today all large-scale facilities have one or more beamlines designed for these types of experiments. For powder X-ray diffraction (PXRD), total scattering (TS), and small-angle X-ray scattering (SAXS), many beamlines use 2D area detectors. The data from these detectors are azimuthally integrated to yield intensity values as a function of scattering angle and, in some cases, as a function of azimuthal angle (*e.g.* Kieffer *et al.*, 2020 ▸; Jensen *et al.*, 2022 ▸; Filik *et al.*, 2017 ▸; Toby & Von Dreele, 2013 ▸). However, while the experimental geometry and data processing are highly similar, today every facility—in many cases even each beamline—produces a distinct file format!

The HDF5 data format (HDF Group, 2026*a* ▸) is widely used for storing raw and, increasingly, processed X-ray data. The benefit of the HDF5 format is that all data and metadata from a whole experiment can be stored in a single file. The caveat, however, is that the data structures in these files vary greatly from facility to facility and even from beamline to beamline. As no standard exists, analysis programs rarely work with these files. Thus, users must use custom programs or scripts to extract the data in the required format. This leaves the user to convert the ‘beamline data format’ to the format used by their chosen refinement program.

Most refinement programs for PXRD work with ASCII-based files for the intensity data. This has traditionally worked well for low data volumes but quickly becomes impractical for increasing data volumes from, for example, µXRD mapping (Christensen *et al.*, 2023 ▸) or XRD-CT (Leemreize *et al.*, 2013 ▸) methods, where hundreds of thousands and even millions of patterns are collected in each experiment. Here, the HDF5 format offers a powerful way to aggregate data in a single file, while also including relevant processing information.

To this end, we at the MAX IV Laboratory (Robert *et al.*, 2023 ▸) and ESRF (Raimondi *et al.*, 2023 ▸) suggest an open data format (NXazint) that is now being used by all beamlines at MAX IV that perform azimuthal integration of area detector scattering data. The format will in the future also be available for use at ESRF beamlines through the EWOKS data processing workflow (De Nolf *et al.*, 2024 ▸). The format is based on HDF5, utilizing the NeXus standard (Könnecke *et al.*, 2015 ▸), and has recently been approved as a set of NeXus application classes. The format is integration software agnostic, thus any integration software can be extended to produce these files. A writer for *pyFAI* (Kieffer *et al.*, 2020 ▸) has been developed and is included since version 2026.02.

The data format is designed for scattering and diffraction data where an azimuthal integration is performed, *e.g.* PXRD, TS, and SAXS. It is designed to include sufficient information about the data processing to enable the azimuthal integration to be reproduced, provided the raw data are available. ‘Conventional’ 1D intensity data as a function of 2θ (or magnitude of the momentum transfer vector, *Q*) and azimuthally binned (2D) data for, for example, texture analysis are supported. Either type of data, or both, can be stored in the file. Errors and/or pixel normalization information can optionally be stored for each dataset above. Auxiliary information and measurements, such as beam intensity and/or transmission data for normalization, can be optionally included if desired for the following analysis.

The data format is intended for direct use in analysis programs, such as Rietveld refinement. Thus, the format, for example, also requires that the wavelength be included as a required entry. Support is already available in *GSAS-II* (Toby & Von Dreele, 2013 ▸), and support is being implemented in upcoming versions of *TOPAS-Academic* (Coelho, 2018 ▸) and *Jana2020* (Petricek *et al.*, 2023 ▸). NXazint files can be explored and visualized by many existing HDF5 tools, including web-based tools, in particular *myHDF5* (HDF Group, 2026*b* ▸). In order to simplify NXazint data visualization even further, we have developed a Python-based viewer, *plaid* (Gjørup, 2026 ▸). This desktop application enables rapid inspection of PXRD data, for example, during beam time. It also offers backwards compatibility by exporting to ASCII files.

Currently, the NXazint format is used at multiple beamlines at MAX IV, and we encourage other beamlines/facilities to adopt it so that it can become the standard data format for this type of experiment. In this way, users can directly repurpose scripts and other resources across different beamlines/facilities. A well documented, standardized format will hopefully also encourage more developers to implement support for it in their analysis/refinement programs.

Funders and large facilities alike are all pushing to implement the FAIR data principles, *i.e.* Findable, Accessible, Interoperable and Reusable data (Wilkinson *et al.*, 2016 ▸). A standardized universal data format for azimuthally integrated data proposed here will help ensure that the data are interoperable and reusable, thus a step towards full FAIR compliance for processed X-ray scattering data. As such, we hope this (standardized) format will help encourage researchers to make the azimuthally integrated data available when publishing. The specification requires that beamline and facility information be included, thereby increasing the traceability of the data.

The NXazint files are not intended to replace the pdCIF format (Toby, 2005 ▸), which is used to report PXRD data and refined structural models. Instead, they are designed as a convenient format for azimuthally integrated experimental data, which often contain many individual patterns that cannot be practically handled as separate text files. We do, however, recommend that authors link to NXazint files when publishing pdCIF files, as shown in Fig. 1 ▸.

The full definition of the format can be found in the official NeXus documentation (NeXus International Advisory Committee, 2026*a* ▸, 2026*b* ▸). Example files are available on the Zenodo repository. Python code examples for reading the NXazint files are available on the *plaid* website (Gjørup, 2026 ▸).

Example file links:

*LaB6.h5*: Minimal NXazint1d file example. https://doi.org/10.5281/zenodo.18685805.

*BTO.h5*: NXazint1d file example, including I0 monitor, sample temperature (not calibrated), and time. https://doi.org/10.5281/zenodo.18685853.

*Magnet.h5*: NXazint2d file example, including I0 monitor, and sample rotation around the vertical axis (omega). https://doi.org/10.5281/zenodo.18685869.

*Magnet_multi.h5*: Combined NXazint1d and NXazint2d file example, including I0 monitor and sample rotation around the vertical axis (omega). https://doi.org/10.5281/zenodo.18685879.

*Magnet_low_res.h5*: Small NXazint2d file example, including I0 monitor, and sample rotation around the vertical axis (omega). https://doi.org/10.5281/zenodo.18685892.

*Pineapple.h5*: NXazint1d mapping file example, including I0 monitor, and sample position. https://doi.org/10.5281/zenodo.18685898.

## Figures and Tables

**Figure 1:**
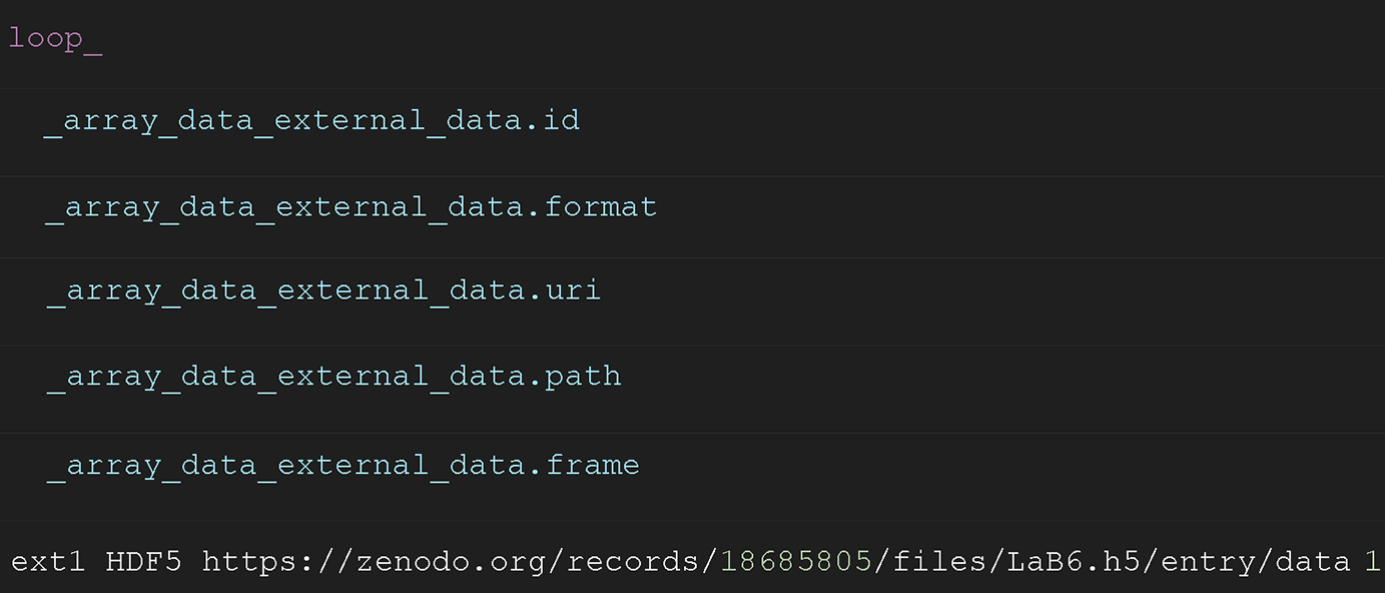
Example concept, inspired by imgCIF, of how to link to the first pattern in an NXazint file archived in the Zenodo repository.
